# nf-core/clipseq - a robust Nextflow pipeline for comprehensive CLIP data analysis

**DOI:** 10.12688/wellcomeopenres.19453.1

**Published:** 2023-07-04

**Authors:** Charlotte West, Charlotte Capitanchik, Chris Cheshire, Nicholas M. Luscombe, Anob Chakrabarti, Jernej Ule

**Affiliations:** 1 The Francis Crick Institute, London, England, UK; 2 Okinawa Institute of Science and Technology Graduate University, Onna, Okinawa, Japan; 3 UK Dementia Research Institute at King's College London, London, UK

**Keywords:** CLIP, Nextflow, RNA-protein interactions, peak calling, reproducible research, bioinformatic pipelines

## Abstract

Crosslinking and immunoprecipitation (CLIP) technologies have become a central component of the molecular biologists’ toolkit to study protein-RNA interactions and thus to uncover core principles of RNA biology. There has been a proliferation of CLIP-based experimental protocols, as well as computational tools, especially for peak-calling. Consequently, there is an urgent need for a well-documented bioinformatic pipeline that enshrines the principles of robustness, reproducibility, scalability, portability and flexibility while embracing the diversity of experimental and computational CLIP tools.

To address this, we present nf-core/clipseq - a robust Nextflow pipeline for quality control and analysis of CLIP sequencing data. It is part of the international nf-core community effort to develop and curate a best-practice, gold-standard set of pipelines for data analysis. The standards enabled by Nextflow and nf-core, including workflow management, version control, continuous integration and containerisation ensure that these key needs are met.

Furthermore, multiple tools are implemented ( *e.g.* for peak-calling), alongside visualisation of quality control metrics to empower the user to make their own informed decisions based on their data.

nf-core/clipseq remains under active development, with plans to incorporate newly released tools to ensure that pipeline remains up-to-date and relevant for the community. Engagement with users and developers is encouraged through the nf-core GitHub repository and Slack channel to promote collaboration. It is available at https://nf-co.re/clipseq.

## Introduction

The capacity to study RNA-protein interactions across the transcriptome was enhanced by the development of crosslinking and immunoprecipitation (CLIP) ^1^. This technology has matured over the past two decades leading to many variants with specific optimisations and use-cases ^2, 3^. At its core, CLIP leverages the capacity of UVC light to form covalent “crosslink” bonds between RNA nucleotides and their bound RNA binding proteins (RBPs). Partial RNase digestion is followed by immunoprecipitation of the RNA-RBP complexes. The RBP is digested away leaving a small polypeptide at the crosslink site. In the latest CLIP technologies, during reverse transcription, the resultant cDNA either truncates ( *e.g.* iCLIP, eCLIP and derivatives ^4– 8^) or incorporates a mutation ( *e.g.* PAR-CLIP and derivatives ^9, 10^) at the crosslinked nucleotide. This key methodological innovation enables the delineation of the crosslink site at individual nucleotide resolution from the reads obtained after high-throughput sequencing.

Importantly, many experimental improvements have made CLIP experiments broadly accessible ^2, 5, 11^. The capacity for sample multiplexing has increased the throughput of these methods ^6^, and improvements in the cDNA library preparation protocols have increased the volume and complexity of CLIP data being generated. As a consequence, there is now more value than ever in systematically integrating one’s results with the wealth of publicly available CLIP data. However, substantial barriers remain to realising the promise of such integrative meta-analysis, as CLIP still requires specialised data processing and analysis approaches ^3, 12, 13^. Thus, there is a great need for easy-to-use, robust, reproducible, scalable and portable analysis pipelines alongside the democratisation of the experimental method.

Moreover, for any CLIP pipeline is it important to consider carefully both quality control assessment and analytic approaches. Quality control assessment of CLIP data is important both at the experimental level ( *e.g.* with visualisation of RNA-protein complexes on the gel) ^2^ and at the computational level ( *e.g.* by assessing library complexity) ^12^. A central aspect of data analysis is peak calling, that is the identification of functionally important or relevant binding sites from either transient interactions or from background noise ^3, 12^, which has been an area of active development in the community since the first CLIP methods. However, there are no *de facto* gold-standard peak calling tools in the field, and often each tool has parameters that need to be tailored to the RBP being studied ^12, 14^. Thus, it would be highly valuable to be able to run multiple tools simultaneously on the same dataset and also assess their results using some simple metrics — particularly for those laboratories without in-house CLIP analysis expertise that have performed experiments to answer a specific biological question.

Although several CLIP pipelines and code walkthroughs are available, they do not meet all of these requirements. PARpipe is specific for the mutation-based PAR-CLIP method ^9^ and is not applicable to the majority of CLIP technologies currently in use ( *e.g.* eCLIP ^7^, iCLIP ^4, 6^, iCLIP2 ^5^) that employ truncation-based approaches to identifying the nucleotide-resolution site of RNA-RBP crosslinking. The iCount software (RRID:SCR_016712) was the first developed for data analysis and peak calling based on cDNA truncation sites ^4, 15^. While it remains effective in identifying functionally relevant sites ^12^, iCount suffers from barriers of portability and scalability. The ENCODE consortium studied the binding of hundreds of RBPs using eCLIP and have published a standard operating procedure for the data processing ^16^, which has been further developed into a pipeline using the Common Workflow Language (available here). However, this is specific for the CLIPper peak-calling tool, which uses whole reads by default, and thus is less applicable to most current CLIP variants that rely on analysis of truncation sites. Very recently, a pipeline called Skipper ^17^, designed for CLIP-based methods that incorporate input libraries into their protocol (such as eCLIP), has been released. This uses Snakemake to ensure scalability, but the lack of containerisation limits its portability to different computational infrastructures. It is also not currently generalisable to datasets without accompanying input libraries as it only implements the Skipper peak-calling approach, which also means that it is non-trivial to perform comparisons with other peak-callers.

Several approaches have also been established to implement CLIP analysis pipelines via web servers, thus making them more accessible to non-bioinformaticians. The first of these was based on the iCount software; more recently CLIP-Explorer ^18^ has been established based on the Galaxy system and includes three peak callers (PEAKachu ^19^, Piranha ^20^, PureCLIP ^14^). While user-friendly, the full underlying source code is not openly available or version-controlled, making it difficult to ensure reproducibility over time and to host on other infrastructures. It is also cumbersome to scale to large numbers of datasets and ultimately difficult for advanced users to customise and implement further new analysis tools as they are developed.

The past two decades have seen the growth of powerful and versatile bioinformatics workflow languages that facilitate the construction, execution, and sharing of intricate, multi-step pipelines ^21^. Baked-in version control, containerisation and resource management empower users to address issues of reproducibility, portability and scalability of bioinformatics analyses. Whilst many options exist, a key factor in choosing to adopt any programming language is the accessibility and activity of the surrounding community. In this regard, Nextflow stands out for the international nf-core project: a “community effort to collect a curated set of analysis pipelines built using Nextflow” ( nf-core ^22^). Within this project, each high-throughput biological data type is represented by a single gold-standard pipeline agreed upon by community consensus. To address the needs for accessible and reproducible CLIP sequencing analysis pipelines, we developed nf-core/clipseq — a pipeline for quality control, pre-mapping, genome mapping, unique molecular identifier (UMI) based PCR deduplication, and multiple peak-calling options, thus implementing best practice guidelines for CLIP data analysis ^3, 12, 13^.

## Methods

### Implementation

The workflow is implemented using the Nextflow workflow language ^23^ as adopted in the nf-core framework ^22^. Through using this well-established template toolkit, we were able to ensure key design principles were instilled from the outset, in particular portability, reproducibility and scalability; continuous integration testing; extensive documentation; and robust versioning and issuing of stable releases.

Importantly, continuous integration is employed using GitHub Actions: a small test dataset is run every time a pull request is made to merge a code branch containing bug fixes or new features to the dev (development) branch. A new version of the pipeline is automatically released when this is merged with the main (stable) branch. Further a full-size test dataset is run on Amazon Web Services (AWS) every time there is a new release.

Furthermore as the pipeline is hosted on nf-core ( here) and was developed to their standards it incorporates extensive online documentation of all the input parameters and output files, with examples from a full-size test dataset run. There is also an active nf-core/clipseq Slack channel where users or other developers can post questions, contribute to further development and can interact directly with the authors and pipeline maintainers.

Development on the nf-core pipeline commenced in November 2020 and the first release was in April 2021. In total there have been 1,870 unique visitors to the repository and 824 unique clones.

### Operation

The pipeline is portable and scalable by design: it can be run on a local computer, high-performance computing cluster (HPC), or a cloud computing infrastructure. However, depending on the organism being studied and the size of its genome, a local computer may not have sufficient memory. For example, for a CLIP experiment in human samples, we would recommend a minimum of 8 cores and 32GB RAM; although an HPC would be advisable for processing multiple samples in parallel. Configuration files for HPCs at many institutes internationally and for cloud services are provided by nf-core ( here) or the user can supply a configuration file with their system’s specification. The minimum software requirements to run the pipeline are Nextflow (at least v20.04.0) and either Conda (RRID:SCR_018317) or a container platform application such as Docker or Singularity.

**Figure 1.**
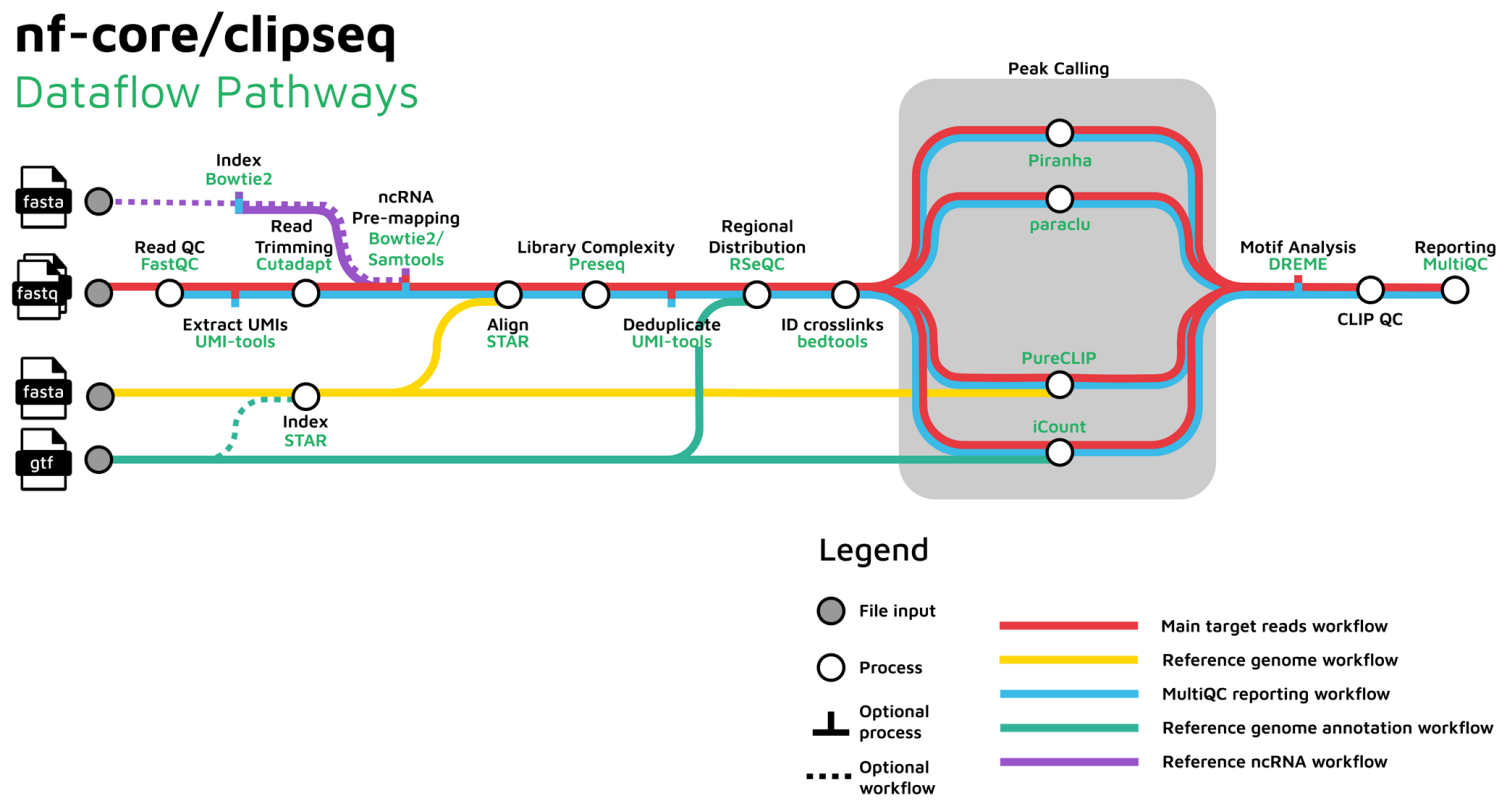
A graphical representation of the nf-core/clipseq workflow. Icons from https://nf-core/sarek; under a CC-BY 4.0 licence.

A graphical representation of the entire workflow is shown in Figure 1. We have implemented a recommended best-practice CLIP analysis pipeline as described in recent reviews ^12, 13^. In the rest of this section, we take the reader through the tools used in each of these steps; some are optional depending on the dataset ( *e.g.* moving the UMI from the sequenced read in the FASTQ to the sequenced read name/header) and some are user-determined ( *e.g.* which peak-calling tool to use).

The starting point for the pipeline is the set of raw, demultiplexed FASTQ files for each sample in the experiment. A standard nf-core sample sheet in comma-separated value format needs to be supplied using the --input argument; this contains the sample name and the path to the associated FASTQ file. Although iGenomes support is included ( --genome) and so many organisms can be specified to automatically link to the necessary sequence and annotation files, it is likely that the user will want specific genome builds or annotations and so instead can supply a genome sequence FASTA file ( --fasta) and transcript annotation GTF file ( --gtf). The annotation can be particularly important for peak calling.

**Figure 2.**
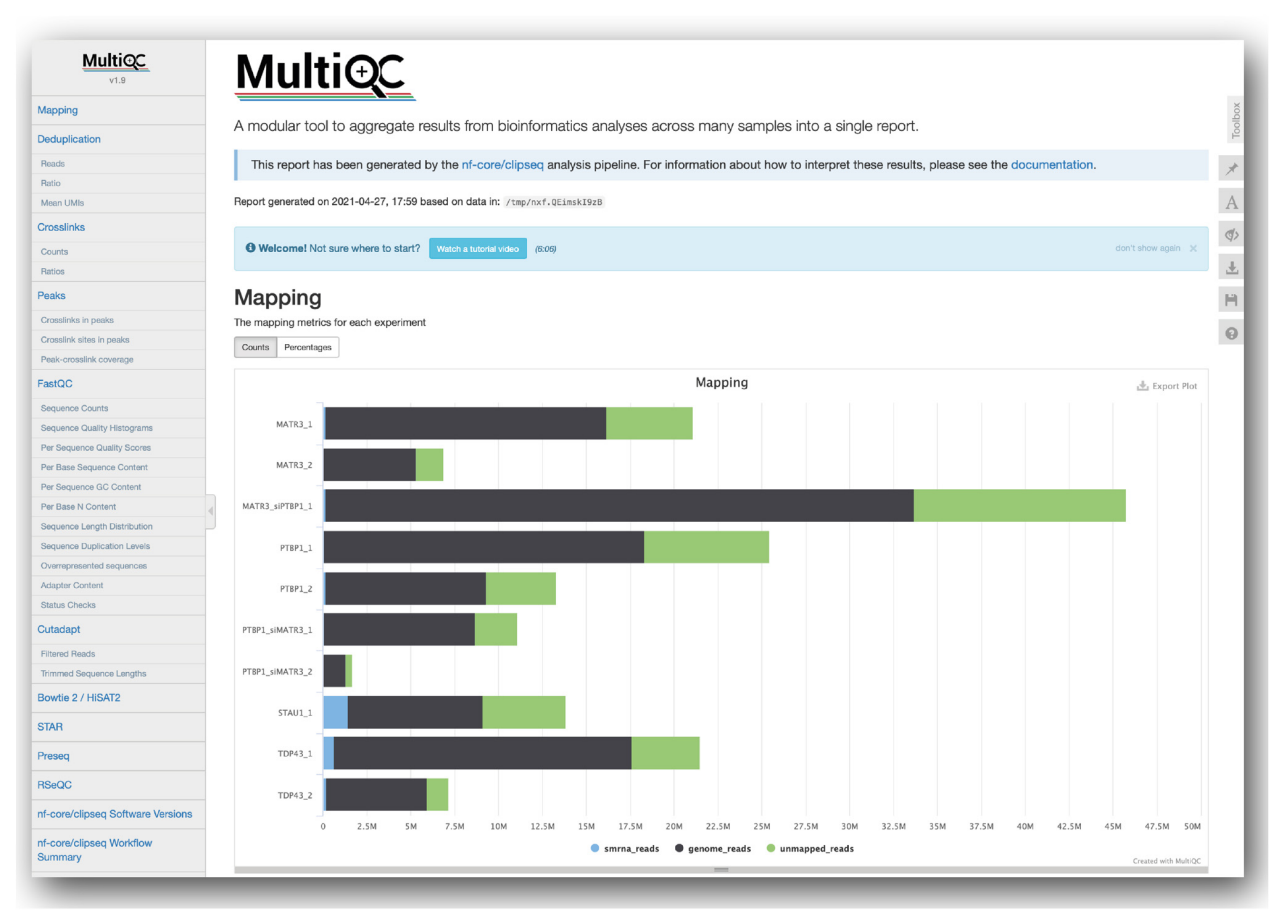
A comprehensive QC report is generated by the pipeline as an interactive HTML document using the MultiQC engine ^31^. This example screenshot of the HTML report from the pipeline run of 10 full-size datasets can be viewed and further explored at https://nf-co.re/clipseq/results.

Next, we describe the primary CLIP data processing workflow. Running in-tandem to this is the CLIP quality control workflow, which we discuss further in “Use cases”. We start by generating alignment indices for the pre-mapping using Bowtie2 ^24^ (RRID:SCR_016368) and genomic alignment using STAR ^25^ (RRID:SCR_004463). We optionally move UMIs to the header of the FASTQ for later deduplication using UMI-tools ^26^ (RRID:SCR_017048) if this has not already been performed by the user as part of demultiplexing before running the pipeline. We then trim sequencing adapters using Cutadapt ^27^ (RRID:SCR_011841) and first pre-map to rRNA and tRNA (discussed further in “Use cases”) and then take the unmapped reads to align to the genome. We collapse PCR duplicates based on read start location and UMI using UMI-tools ^26^ and then identify the crosslink coordinate using BEDTools ^28^ (RRID:SCR_006646). BedGraph files of the crosslink events are also generated to facilitate visualisation in a genome browser. The crosslink events are then used as input to the peak calling tools: iCount ^15^, paraclu ^29^, Piranha ^20^ (RRID:SCR_010903) and PureCLIP ^14^ (also discussed further in “Use cases”). The peaks defined by each can then be used by DREME ^30^ (RRID:SCR_001783) to identify short enriched motifs. A summary of the workflow run, parameter arguments and software versions are included alongside all the analysis and quality control (QC) results as part of the summary HTML ( Figure 2).

The workflow is highly customisable with many other well-documented parameters ^32^. However, they all have sensible default values, to enable easy running out-of-the-box. These can be overridden by the user as part of the run command. Moreover, due to the integration with nf-core, this run command can either be written out manually or generated using the nf-core launch graphical user interface (GUI).

## Use cases

As discussed earlier, as part of the development process, a test run of 10 full-sized published iCLIP datasets (for the RBPs MATR3, PTBP1, STAU1 and TDP-43 ^33– 35^, see “Source data”) is launched on AWS on release of a new pipeline version. All the results and all the outputs from the run of the pipeline on the use case are available as “Underlying data” ^36^ and on the website ( here) so the user can see a complete example of the results folder structure and output files. In this section, we first explain how to run the pipeline with these datasets to reproduce these results as an example of the pipeline’s operation. Then, we discuss in further detail three key sections of the CLIP analysis workflow: i) pre-mapping to non-coding RNA; ii) peak calling; and iii) CLIP QC assessment.

### Processing CLIP sequencing data using nf-core/clipseq

After downloading the FASTQ files from the Sequence Read Archive, and the reference genome files from GENCODE, we prepare a comma-separated value (csv) samplesheet file specifying the sample names and the paths to the raw FASTQ files downloaded from the Sequence Read Archive onto the user’s system following the template:


sample,fastq
TDP43_1,/path/to/data/ERR1530360.fastq.gz
TDP43_2,/path/to/data/ERR1530361.fastq.gz
...


Next we download the desired version of the pipeline:


nextflow pull nf-core/clipseq -r 1.0.0


And then we can run the pipeline using:


nextflow run nf-core/clipseq -r 1.0.0 \
-profile crick \
--input samplesheet.csv \
--smrna_org human \
--fasta GRCh38.primary_assembly.genome.fa.gz \
--gtf gencode.v37.primary_assembly.annotation.gtf.gz \
--move_umi 'NNNNNNNNN' \
--peakcaller 'icount,paraclu,pureclip,piranha' \
--pureclip_iv 'chr1;chr2' \
--motif true


Here we have run the pipeline using our institution’s profile ( -profile crick) and as discussed in “Operation”, this should be replaced with the user’s institution's configuration file, or as a minimum either Conda, Docker or Singularity. As our datasets are from human cell line experiments, we specify the use of the human pre-mapping files contained within the pipeline ( --smrna_org). These CLIP experiments made use of 9 nucleotide UMIs during the experimental protocol, which are contained within the raw FASTQ files and need to be extracted and properly handled ( --move_umi). We use all four peak callers that are included ( --peakcaller). For this particular use case, to reduce the run time and memory requirements, we specify that the PureCLIP peak caller will only use chromosomes 1 and 2 to learn the parameters of its Hidden Markov Model as discussed in their documentation ( --pureclip_iv). Finally, we specify the pipeline to examine the peaks for motifs ( --motif).

This pipeline run will output 20 folders containing all the results (see “Underlying data” or the website here). The main folders of interest to the majority of users are: i) pipeline_info (containing details of the pipeline execution, runtime and resource usage); ii) multiqc (containing the QC analysis discussed below); iii) dedup (containing BAM files of read alignments after collapsing PCR duplicates); iv) xlinks (containing BED and BEDGraph files of the crosslink site positions and counts); v) one folder for each peak caller that was run containing BED files of the peak positions; and vi) one folder for each peak caller that was run containing the discovered motifs (if any).

The outputs of the pipeline will form the basis for subsequent tailored analysis based on the user’s biological questions. This could include using the BED and BEDGraph files for visualisation in an genome browser such as the Integrative Genomics Viewer ( IGV) ^37^ (RRID:SCR_011793) or for comparative visualisation using *clipplotr* ^38^; or the peak files for generating RNA maps with orthogonal sequencing data ^39– 41^ or alternative motif analysis approaches e.g. PEKA ^42^, kp-Logo ^43^ or HOMER ^44^ (RRID:SCR_010881).

### Pre-mapping to non-coding RNA

It is usually recommended to pre-map to non-coding RNA (ncRNA) sequences (especially rRNA and tRNA) before proceeding to genomic or transcriptomic mapping ^12, 16^. This is because these sequences are present in multiple similar copies throughout the genome and thus often sequencing reads cannot be uniquely assigned to a specific locus. Hence, if one is interested in binding to rRNA or to tRNAs, directly mapping to these sequences is required. For tRNA it is also useful to group these into iso-acceptor sub-families ^16, 45, 46^. Furthermore, tRNA and other ncRNA sequences can occur within genes, particularly introns, and without due consideration this can result in the misassignment of sequencing reads to an mRNA instead of the ncRNA ^47^.

We provide curated FASTA files for pre-mapping to human, mouse, rat, zebrafish, fruit fly and yeast ncRNA that can be called simply using *e.g.* --smrna_org human. If iGenomes is being used for one of these organisms, then this argument is automatically supplied. The curated files can also be found in the pipeline repository. Alternatively, the user can supply their custom pre-mapping sequences using --smrna_fasta.

### Peak calling

Peak calling is a central step in CLIP analysis ^3, 12, 13, 48^. For protocols with stringent purification and experimental quality control steps, peak calling enables the delineation of high occupancy binding sites from those with more transient binding — the former are more likely to represent functionally relevant interactions. Some researchers advocate the use of input controls, for example eCLIP’s “size-matched input” control, however recent findings suggest that any use of input control should be carefully evaluated as the control sample can include foreground signal and does not necessarily increase data specificity, while its use can greatly decrease the number of identified CLIP peaks ^3, 49^.

A plethora of peak calling tools have been developed over the years and new ones continue to be released ^3, 12^. Here, in nf-core/clipseq we have not been prescriptive in the choice of peak-caller and leave this to the user to determine. We have implemented four different tools: iCount ^4^, paraclu ^29^, Piranha ^20^ and PureCLIP ^14^, any combination of which can be run in parallel as part of the pipeline. To assist in the choice of peak-caller simple quality control metrics are generated that the user can review to compare results. For more detailed assessment, we recommend further analysis outside of the scope of this pipeline, including integration with orthogonal data, using for example RNA maps ^12^. Often peak calling parameters for a given tool benefit from being tuned to the RBP under study ^12, 18^. For example, for iCount and Piranha, an RBP such as FUS with a more distributed binding preference will require a wider window size, whereas an RBP such as HuR/ELAVL1 with more punctate binding will need a narrower window size ^12^.

### CLIP QC assessment

We have implemented both general RNA sequencing QC tools and specific CLIP sequencing metrics relevant for assessing peak-calling. All of these results are collated, summarised and presented in an interactive HTML document using MultiQC ^31^ (RRID:SCR_014982) ( Figure 2).

In terms of general RNA sequencing metrics, we summarise the logs from raw read assessment using FastQC ^50^ (RRID:SCR_014583); adapter trimming using Cutadapt ^27^; pre-mapping using Bowtie2 ^24^; and genomic alignment using STAR ^25^. Additionally, we provide a customised overview of the mapping metrics: how many reads aligned to the pre-mapping (ncRNA) index, how many aligned uniquely to the genome, and how many remained unaligned. Mapping metrics can flag both technical issues in pipeline running ( *e.g.* low genome alignment, which can represent incorrect genome input) and experimental issues ( *e.g.* in the case of a known mRNA-binding protein mapping predominantly to ncRNAs). The regional distribution of aligned reads over genome features is assessed using RSeQC ^51^ (RRID:SCR_005275).

As CLIP-specific metrics, first, we calculate three metrics related to UMI-based deduplication: the number of reads before and after deduplication; this presented as a ratio; and the mean number of UMIs per position for each experiment (as calculated by UMI tools ^26^). A large reduction in reads after deduplication, and therefore a correspondingly high deduplication ratio, can indicate poor library complexity that can result from experimental issues, such as poor protein-RNA crosslinking. Additionally the complexity of a sequencing library is estimated using Preseq ^52^ (RRID:SCR_018664). This can give an indication of the number of redundant reads and whether additional sequencing of the sample would yield additional complexity. Then, we show the total number of genomic crosslink events (number of cDNAs) for each experiment and the number of crosslink sites ( *i.e.* unique genomic loci with crosslink events) and we also present this as a ratio. Finally, we generate three metrics to assess each peak calling tool and enable comparisons across them: i) the total percentage of crosslink events within peaks; ii) the total percentage of crosslink sites within peaks; and iii) the total percentage of nucleotides within peaks that are covered by a crosslink site. We intend these metrics to be analogous to those used in ChIP-seq to assess the specificity of the data, and also to assess the performance of peak callers for each given sample.

## Conclusions

In conclusion, we have presented the nf-core/clipseq pipeline for the analysis of CLIP data produced by a range of CLIP technologies. This addresses a need for a robust, reproducible, scalable and portable pipeline implementing best-practice analysis guidelines. The inclusion of multiple peak calling tools and extensive QC reporting enables the informed parameterisation of the peak callers and confidence in the biological interpretation of results. It remains under active development and future features planned to be incorporated as part of a transition to Nextflow DSL2 include transcriptome mapping, recently released peak-callers and more CLIP-focused motif analysis tools. We hope that it is useful to both novice and advanced users of CLIP data, and that nf-core/clipseq provides a platform to foster collaboration; we welcome the broader CLIP analysis community to contribute to its ongoing development and to keep it state of the art.

## Data Availability

Raw demultiplexed FASTQ files for the CLIP experiments processed in “Use cases” are publicly available from the Sequence Read Archive under accessions: ERR1530360, ERR1530361, ERR605258, ERR2210802, ERR2210803, ERR2210804, ERR2214592, ERR2214593, ERR2214594, ERR2214595. Zenodo: Underlying data for: nf-core/clipseq - a robust Nextflow pipeline for comprehensive CLIP data analysis. https://doi.org/10.5281/zenodo.7971140 ^36^. This project contains the following underlying data: nf_core_clipseq_testrun.tar.gz (Processed outputs from the use case presented). Data are available under the terms of the Creative Commons Attribution 4.0 International license (CC-BY 4.0).

## Software availability

**Software available from:** https://nf-co.re/clipseq

**Source code available from:** https://github.com/nf-core/clipseq

**Archived source code as at time of publication:** https://doi.org/10.5281/zenodo.4723017 ^32^.

**Licence:** MIT
