# Supplementary material for: nf-core/clipseq - a robust Nextflow pipeline for comprehensive CLIP data analysis
Source: Wellcome Open Res. 2023 Jul 4;8:286. [Version 1] doi: 10.12688/wellcomeopenres.19453.1 (PMC10565428; doi:10.12688/wellcomeopenres.19453.1)
Supplement: Peer review history

# Reviewer report: Reviewer response for version 1

**DOI:** 10.21956/wellcomeopenres.21549.r67585

**Published:** 2023-10-10

**Authors:** Emmanouil Maragkakis

**Affiliations:** 1 Laboratory of Genetics and Genomics, National Institute on Aging, Baltimore, Maryland, USA

In this work the authors present a pipeline implemented in Nextflow for the analysis of CLIP-Seq datasets. CLIP-Seq is widely used to capture RNA-binding protein interactions with their target genes. However, the analysis of CLIP-Seq datasets can be challenging especially for non-experts in the field. The development of pipelines, besides facilitating analysis, provide the added benefit of increased reproducibility and correctness. Overall, the tool is nicely described, and the pipeline is well designed. Nextflow and nf-core are stable platforms that facilitate customization and deployment on diverse hardware environments. It will be important that the authors maintain and support the software in the long-term. This tool will be helpful to the community, will facilitate the analysis of CLIP-Seq data and will increase research reproducibility.

Are the conclusions about the tool and its performance adequately supported by the findings presented in the article?

Yes

Is the rationale for developing the new software tool clearly explained?

Yes

Is the description of the software tool technically sound?

Yes

Are sufficient details of the code, methods and analysis (if applicable) provided to allow replication of the software development and its use by others?

Yes

Is sufficient information provided to allow interpretation of the expected output datasets and any results generated using the tool?

Yes

Reviewer Expertise:

Bioinformatics, genomics, RNA

I confirm that I have read this submission and believe that I have an appropriate level of expertise to confirm that it is of an acceptable scientific standard.

# Reviewer report: Reviewer response for version 1

**DOI:** 10.21956/wellcomeopenres.21549.r67581

**Published:** 2023-10-10

**Authors:** Shiyong Liu

**Affiliations:** 1 Huazhong University of Science and Technology, Wuhan, Hubei, China

CLIP-based technologies produced lots of protein-RNA binding site data, which is important for RNA biology. However, an easy and better computational CLIP tool is still needed. The authors developed nf-core/CLIPseq-a robust Nextflow pipeline for quality control and analysis of CLIP sequencing data. They claim it is a best-practice, gold-standard set of pipelines for data analysis. It is really good and friendly for those people who is interested in RBP biology. My concern comments are the following.

1. For peak-calling, what are the benchmarking results for PARpipe, Skipper, iCount, CLIP-Explorer, and other three peak callers (PEAKachu, Piranha, PureCLIP)? As a user, most researcher are caring about which peak callers is better?
2. The authors did a test run of 10 full-sized published iCLIP dataset (for the RBPs MATR3, PTBP1, STAU1 and TDP-43). What is the accuracy for these tools on different RBPs? The authors proposed three metrics to assess each peak calling tool and enable comparisons across them. So, what is the benchmarking result for comparison? What is the gold answer for each problem (for example, Peaker calling)? Could the authors rank these tools ?

Are the conclusions about the tool and its performance adequately supported by the findings presented in the article?

Partly

Is the rationale for developing the new software tool clearly explained?

Yes

Is the description of the software tool technically sound?

Yes

Are sufficient details of the code, methods and analysis (if applicable) provided to allow replication of the software development and its use by others?

Yes

Is sufficient information provided to allow interpretation of the expected output datasets and any results generated using the tool?

Partly

Reviewer Expertise:

RNA-binding protein,  RNA-protein interaction

I confirm that I have read this submission and believe that I have an appropriate level of expertise to confirm that it is of an acceptable scientific standard, however I have significant reservations, as outlined above.

# Reviewer report: Reviewer response for version 1

**DOI:** 10.21956/wellcomeopenres.21549.r67582

**Published:** 2023-10-03

**Authors:** Yucheng Yang

**Affiliations:** 1 Fudan University, Shanghai, Shanghai, China

Given the recent advancements in CLIP-seq technologies and their broad applications, it is imperative to develop computational tools for the uniform processing and peak calling of CLIP-seq datasets. This work aims to provide a pipeline for the quality control and analysis of CLIP-seq data. This idea is not new. The only method innovation from this work is the construction of the project website ( https://nf-co.re/clipseq) such that the usage of the pipeline is quite easy. As a summary, this work may add to the literature of computational tools of CLIP-seq data if the following concerns are clarified.

 Major concerns:

 CLIP-seq technologies include a variety of CLIP-seq experimental assays, including HITS-CLIP, PAR-CLIP, iCLIP and eCLIP. For each type of CLIP-seq assays, there have been several computational tools for the data quality control and peak calling, e.g., CIMS/PEAKachu/CLIPper/CTK/Piranha for HITS-CLIP related technologies (HITS-CLIP, BrdU-CLIP), PARalyzer/Mi-Clip/CTK/Piranha for PAR-CLIP, CITS/PureCLIP/CTK/Piranha for iCLIP related technologies (iCLIP, eCLIP, iCLAP, urea-iCLIP, 4sU-iCLIP, Fr-iCLIP), and CLIPper/CTK/Piranha for eCLIP. Notably, some of these tools are developed for a specific CLIP-seq assay, while some (e.g., Piranha and CTK) can be applied to different CLIP-seq assays.

 The authors may provide a more comprehensive introduction to the principles and characteristics for these existing computational tools in the main text.

 The authors may need to implement more computational tools (mentioned above) in their pipeline. More importantly, it should be made clear that which tool(s) could be used for which kind of CLIP-seq assay.

 Finally, the overlap of the peaks identified by using different tools from the same dataset should be shown in the main figure.

 Minor concerns:

 “e.g.” should be “e.g.,”

 “i.e.” should be “i.e.,”

Are the conclusions about the tool and its performance adequately supported by the findings presented in the article?

Partly

Is the rationale for developing the new software tool clearly explained?

Yes

Is the description of the software tool technically sound?

Yes

Are sufficient details of the code, methods and analysis (if applicable) provided to allow replication of the software development and its use by others?

Yes

Is sufficient information provided to allow interpretation of the expected output datasets and any results generated using the tool?

Yes

Reviewer Expertise:

Bioinformatics

I confirm that I have read this submission and believe that I have an appropriate level of expertise to confirm that it is of an acceptable scientific standard, however I have significant reservations, as outlined above.
